# Thermoelectric Performance of Non-Stoichiometric Permingeatite Cu_3+m_SbSe_4_

**DOI:** 10.3390/ma17174345

**Published:** 2024-09-02

**Authors:** DanAh Kim, Il-Ho Kim

**Affiliations:** Department of Materials Science and Engineering, College of Engineering, Korea National University of Transportation, Chungju 27469, Republic of Korea; rlaeksdk0813@naver.com

**Keywords:** permingeatite, thermoelectric, non-stoichiometric, off-stoichiometric

## Abstract

Non-stoichiometric permingeatites Cu_3+m_SbSe_4_ (−0.04 ≤ m ≤ −0.02) were synthesized, and their thermoelectric properties were examined depending on the Cu deficiency. Phase analysis by X-ray diffraction revealed no detection of secondary phases. Due to Cu deficiency, the lattice parameters of tetragonal permingeatite decreased compared to the stoichiometric permingeatite, resulting in a = 0.5654–0.5654 nm and c = 1.1253–1.1254 nm, with a decrease in the c/a ratio in the range of 1.9901–1.9903. Electrical conductivity exhibited typical semiconductor behavior of increasing conductivity with temperature, and above 423 K, the electrical conductivity of all samples exceeded that of stoichiometric permingeatite; Cu_2.96_SbSe_4_ exhibited a maximum of 9.8 × 10^3^ Sm^−1^ at 623 K. The Seebeck coefficient decreased due to Cu deficiency, showing p-type semiconductor behavior similar to stoichiometric permingeatite, with majority carriers being holes. Thermal conductivity showed negative temperature dependence, and both electronic and lattice thermal conductivities increased due to Cu deficiency. Despite the decrease in the Seebeck coefficient due to Cu deficiency, the electrical conductivity increased, resulting in an increase in the power factor (especially a great increase at high temperatures), with Cu_2.97_SbSe_4_ exhibiting the highest value of 0.72 mWm^−1^K^−2^ at 573 K. As the carrier concentration increased due to Cu deficiency, the thermal conductivity increased, but the increase in power factor was significant, with Cu_2.98_SbSe_4_ recording a maximum dimensionless figure-of-merit of 0.50 at 523 K. This value was approximately 28% higher than that (0.39) of stoichiometric Cu_3_SbSe_4_.

## 1. Introduction

Thermoelectric materials are promising alternative energy materials in that they can directly convert waste heat into electrical energy [1,2]. The performance of thermoelectric materials is evaluated by the dimensionless figure of merit (ZT), defined as ZT = α^2^σκ^−1^T, where α^2^σ is the power factor (α is the Seebeck coefficient, σ is the electrical conductivity), κ is the thermal conductivity, and T is the absolute temperature [3,4]. To increase the ZT value, the power factor of the thermoelectric material must be increased while reducing the thermal conductivity [1,5]. Unlike the Bi−Te and Pb−Te thermoelectric compounds currently in use, Cu−Sb−S and Cu−Sb−Se chalcogenides are attracting attention as economically and eco-friendly materials: skinnerite Cu_3_SbS_3_ [6], bytizite Cu_3_SbSe_3_ [7], famatinite Cu_3_SbS_4_ [8], permingeatite Cu_3_SbSe_4_ [9], tetrahedrite Cu_12_Sb_4_S_13_ [10], hakite Cu_12_Sb_4_Se_13_ [11], chalcostibite CuSbS_2_ [12], and pribramite CuSbSe_2_ [13]. Among these materials, permingeatite (Cu_3_SbSe_4_; space group I$\bar{4}$2m) has a structure similar to the modified zinc blende (ZnS; space group F43m) [2]. This compound is an appropriate choice for a p-type thermoelectric material at moderate temperatures owing to its narrow band-gap energy (0.29–0.4 eV) and high carrier effective mass (≈1.1 me) [14,15,16].

Research efforts are underway to maximize the power factor by controlling carrier concentration through doping, which involves partially substituting certain elements into the Cu/Sb/Se sites of permingeatite, while simultaneously reducing the thermal conductivity through lattice scattering [16,17,18]. Doping the Cu site requires lowering the Cu chemical potential, which can lead to an increase in the formation of Cu vacancies. These vacancies negate the effects of intentional dopants like Mg or Zn, possibly contributing to the challenges in achieving n-type doping in Cu_3_SbSe_4_ [2]. However, there are few reports on non-stoichiometric studies regarding the adjustment in Cu content. Control of chemical composition in stoichiometry can influence the physical properties of semiconductors [19]. According to Wei et al. [20], Cu-deficient permingeatite Cu_3–x_SbSe_4_ (0 ≤ x ≤ 0.075) could increase charge carriers (holes), leading to an increase in electrical conductivity. Kwak and Kim [19] also found the changes in thermoelectric properties through Cu content adjustment in tetrahedrite (Cu_12+m_Sb_4_S_13_; −0.04 ≤ m ≤ 0.04); excess Cu reduced the thermal conductivity due to additional phonon scattering, while Cu deficiencies (vacancies) provided additional charge carriers, improving electrical properties. They achieved a maximum ZT value of 0.91 at 723 K for Cu_11.9_Sb_4_S_13_ (improved from a ZT of 0.86 for stoichiometric tetrahedrite). In this study, Cu-deficient permingeatites and Cu_3+m_SbSe_4_ (−0.04 ≤ m ≤ −0.02) were prepared to investigate the influence of non-stoichiometry on the thermoelectric performance.

## 2. Experimental Procedure

Non-stoichiometric Cu_3+m_SbSe_4_ (m = −0.02, −0.03, and −0.04) were synthesized via mechanical alloying (MA) using elemental powders of Cu, Sb, and Se with high-purity (99.9–99.999%). MA was conducted at 350 rpm for 12 h in an Ar atmosphere within the stainless-steel container. The synthetic powder was then subjected to hot pressing (HP) at 573 K for 2 h under 70 MPa in a vacuum. The optimal process conditions of MA−HP for permingeatite were determined in our previous studies [15,16].

The phases and lattice parameters of the synthesized specimens were analyzed using X-ray diffraction with Cu Kα radiation (D8-Advance, Bruker, Billerica, MA, USA) and Rietveld refinement (TOPAS, Bruker). The microstructure of the sintered pellets was observed using the backscattered electron mode of a scanning electron microscope (Quanta400, FEI, Lausanne, Switzerland). The Hall coefficient, carrier concentration, and carrier mobility were evaluated at room temperature using the Hall effect measurement instrument (Keithley 7065). Electrical conductivity and Seebeck coefficient were measured using the ZEM-3 system (Advance Riko, Yokohama, Japan). The thermal diffusivity was evaluated using a TC-9000H equipment (Advance Riko), and then the thermal conductivity was assessed using the measured density of the specimen and theoretical specific heat (0.32 Jg^−1^K^−1^) [21]. Power factor and ZT values were calculated based on the above thermoelectric parameters obtained in the temperature range of 323–623 K. Comparison was made with the thermoelectric characteristics of stoichiometric Cu_3_SbSe_4_ prepared using the same process [21].

## 3. Results and Discussion

**Figure 1:**
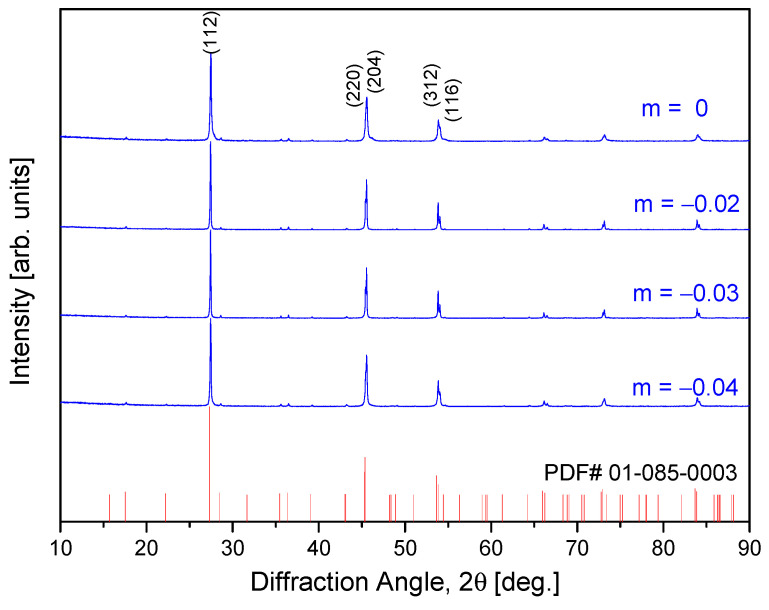
X-ray diffraction patterns of non-stoichiometric permingeatite Cu_3+m_SbSe_4_ prepared by the MA−HP process.

Figure 1 shows the X-ray diffraction patterns of non-stoichiometric Cu_3+m_SbSe_4_ produced via MA−HP. All diffraction peaks matched the standard diffraction data of tetragonal permingeatite (PDF# 01-085-0003), and no secondary phase was identified. However, Kumar et al. [22] observed the presence of small amounts of secondary phases in the diffraction peaks between 30° and 60° for the Cu_2.96_SbSe_4_ sample prepared using vacuum melting, followed by pulverizing and spark plasma sintering. This indicates that the preparation method combining MA and HP in this study is a practical and effective way for the synthesis of non-stoichiometric permingeatite compounds. Compared to the lattice constants of stoichiometric Cu_3_SbSe_4_ (a = 0.5661 nm and c = 1.1280 nm), both a- and c-axes were reduced due to Cu deficiency (a = 0.5654–0.5655 nm and c = 1.1253–1.1254 nm). Additionally, the tetragonality (c/a ratio) of the lattice decreased from 1.9926 to 1.9901–1.9903. Wei et al. [20] reported decreases in lattice constants of Cu_3–x_SbSe_4_ (x = 0–0.075) from a = 0.5655 nm and c = 1.1253 nm to a = 0.5651 nm and c = 1.1248 nm due to a decrease (deficiency) in Cu content; however, when x is 0.075, the XRD diffraction peaks shift to lower angles, and no further reduction in lattice constants is observed. Kwak and Kim [19] discovered that for non-stoichiometric cubic tetrahedrite Cu_12+m_Sb_4_S_13_ (−0.3 ≤ m ≤ 0.3), the lattice constant decreased from a = 1.0350 nm (at m = 0) to a = 1.0338 nm for Cu-poor tetrahedrites at m = −0.3, while increased to a = 1.0384 nm for Cu-rich tetrahedrites at m = 0.3.

**Figure 2:**
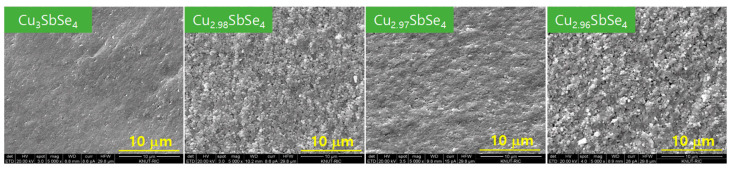
Micrographs of non-stoichiometric permingeatite Cu_3+m_SbSe_4_.

Figure 2 displays microstructures of Cu-deficient permingeatite observed using scanning electron microscopy. They contained some porosity, but significant changes in microstructure due to Cu vacancies were not observed. Compared to the theoretical density (5.86 gcm^−3^) of Cu_3_SbSe_4_ with stoichiometric composition [23], the relative densities of all specimens were in the range of 96.5–98.1%, as shown in Table 1. All MA−HP specimens exhibited a well-crystallized morphology with an average crystallite size of 78 nm. The major fracture mode for Cu-deficient permingeatite was intergranular fracture, which is common for materials with fine grains. Wei et al. [20] also discovered the same morphology as our fractured specimens but found that Cu-content-modified grain growth in non-stoichiometric samples with Cu deficiency results in larger grain sizes compared to stoichiometric samples. However, in this study, no significant change in grain size due to Cu deficiency was observed.

**Figure 3:**
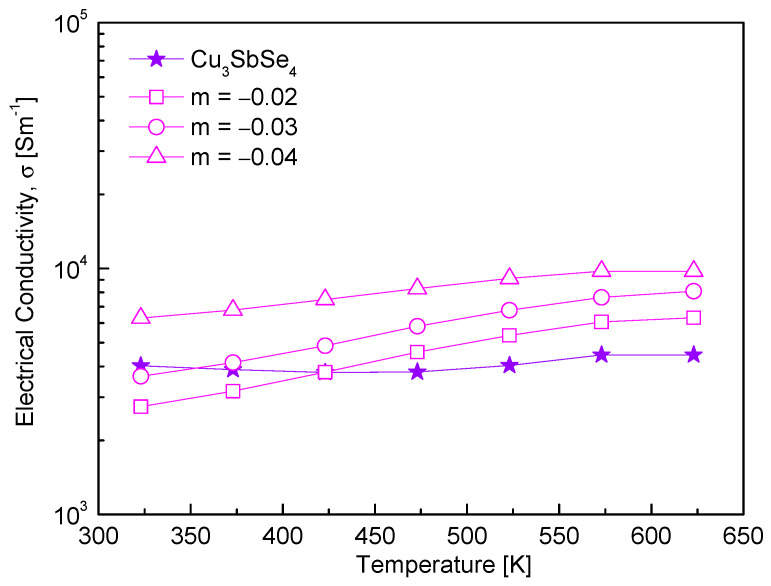
Electrical conductivity of non-stoichiometric permingeatite Cu_3+m_SbSe_4_.

Figure 3 shows the electrical conductivity of Cu_3+m_SbSe_4_. As the Cu deficiency increased, the electrical conductivity increased. Compared to Cu_3_SbSe_4_, the electrical conductivity was higher at temperatures above 423 K. The nondegenerate nature of the electrical transport was not affected by the Cu deficiencies. In the temperature range of 323–623 K, the electrical conductivity increased from (4.2–4.5) × 10^3^ Sm^−1^ for Cu_3_SbSe_4_ [16] to (6.3–9.8) × 10^3^ Sm^−1^ for Cu_2.96_SbSe_4_. This was because the Cu deficiency increased the charge carrier (hole) concentration. It is well understood that even minor deviations from stoichiometric chemical composition can influence the physical properties of semiconductors. Specifically, deficiencies in Cu can introduce extra holes, thereby increasing carrier concentration and enhancing electrical conductivity [24,25]. According to Kwak and Kim [19], as the Cu deficiency increased in Cu_12+m_Sb_4_S_13_ (−0.3 ≤ m ≤ 0.3), the hole concentration increased, leading to an increase in electrical conductivity, while the excess Cu contributed to lowering the carrier concentration. Xia et al. [26] also found in Cu_1−x_InTe_2_ (0 ≤ x ≤ 0.10) that the Cu deficiency increased the carrier concentration from 2 × 10^18^ to 3 × 10^18^ cm^−3^ and decreased the mobility from 100 to 40 cm^−2^V^−1^s^−1^.

As shown in Table 1, the Cu deficiency in permingeatite increased the carrier concentration from 5.2 × 10^18^ to (7.9–9.6) × 10^18^ cm^−3^ while decreasing the mobility from 505 to 210–410 cm^−2^V^−1^s^−1^. The carrier concentration increased with greater Cu deficiency, which was consistent with the observed changes in lattice constants with varying Cu deficiencies. Since both carrier concentration and lattice constants reflect the extent of artificially introduced Cu deficiencies, it can be concluded that Cu deficiencies have been intentionally introduced and have influenced the structure and properties of the permingeatite compounds. The carrier mobility in the non-stoichiometric samples was lower compared to the stoichiometric sample. This suggests that point defects resulting from Cu deficiencies affect the carrier scattering mechanism. Generally, as the carrier concentration increases, the mobility decreases. However, in the case of the specimen with m = −0.04, the mobility increased despite the increase in carrier concentration. Although we cannot provide a definitive explanation for this, changes in lattice parameters (an increase in the c/a axial ratio) and an increase in sintering density (relative density) may be contributing factors.

Do et al. [2] found that a single Cu vacancy in the unit cell does not significantly alter the band structure; energy states near the valence band maxima remain largely unaffected, and there is only a small splitting of the conduction band minima. This suggests that the frequently observed p-type behavior in as-prepared permingeatite can be attributed to native Cu vacancies. This has been experimentally confirmed by Wei et al. [20], who controlled the hole concentration by adjusting the Cu deficiency.

**Figure 4:**
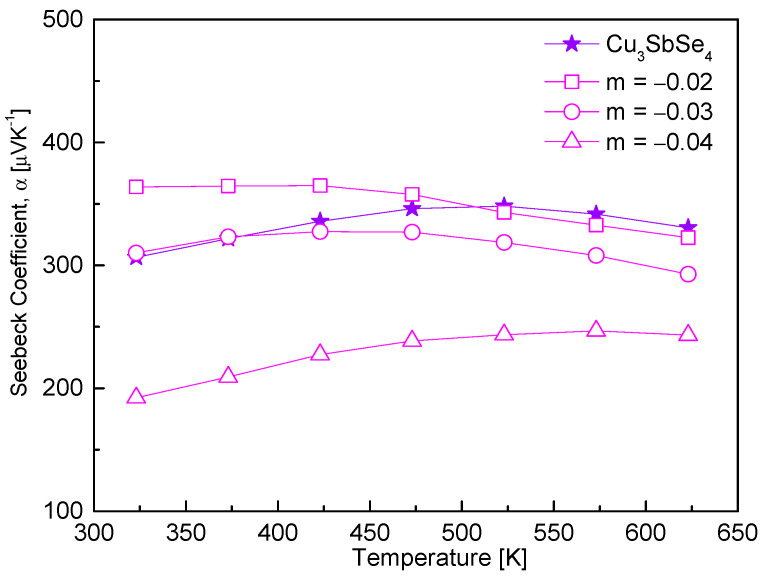
Seebeck coefficient of non-stoichiometric permingeatite Cu_3+m_SbSe_4_.

Figure 4 represents the Seebeck coefficient of Cu_3+m_SbSe_4_. All samples exhibited positive Seebeck coefficient values, which indicate p-type semiconductor behavior. Do et al. [2] modeled permingeatite as a periodic supercell and calculated the formation energies of various defects. They found positive formation energies of vacancies with values increasing from Cu (0.65 eV) to Se (0.94 eV) to Sb (2.13 eV); hence, forming vacancies at any atomic site requires energy. Among these, only Cu vacancies act as acceptors, while Se vacancies do not seem to contribute charge carriers. The results also indicate that the observed p-type behavior in nominally pure Cu_3_SbSe_4_ is likely due to the presence of Cu vacancies rather than Se vacancies. In this study, due to the deficiency of Cu, the Seebeck coefficient decreased, resulting from the increase in carrier concentration. Assuming a single parabolic band for carriers near the Fermi level, the Seebeck coefficient for nondegenerate semiconductors can be expressed as a function of the carrier concentration [20]. In this case, the Seebeck coefficient is inversely proportional to the carrier concentration. Cu_2.98_SbSe_4_ exhibited a Seebeck coefficient ranging from 363 to 322 μVK^−1^ at temperatures from 323 to 623 K, while Cu_2.96_SbSe_4_ exhibited lower values of 192–243 μVK^−1^. Stoichiometric Cu_3_SbSe_4_ demonstrated 307–348 μVK^−1^ in the same temperature range [16]. Skoug et al. [27] reported 300–400 μVK^−1^ at 80–623 K for undoped permingeatite. Wei et al. [20] discovered that all samples of Cu_3−x_SbSe_4_ (0 ≤ x ≤ 0.075) exhibited values higher than 320 μVK^−1^ at 323–623 K. Kumar et al. [22] observed a decreasing trend in the Seebeck coefficient with increasing temperature and Cu deficiency for all samples of Cu_3−δ_SbSe_4_ (0 ≤ δ ≤ 0.04) in the range of 300–675 K, with Cu_2.99_SbSe_4_ exhibiting a maximum value of 263 μVK^−1^ at 475 K.

**Figure 5:**
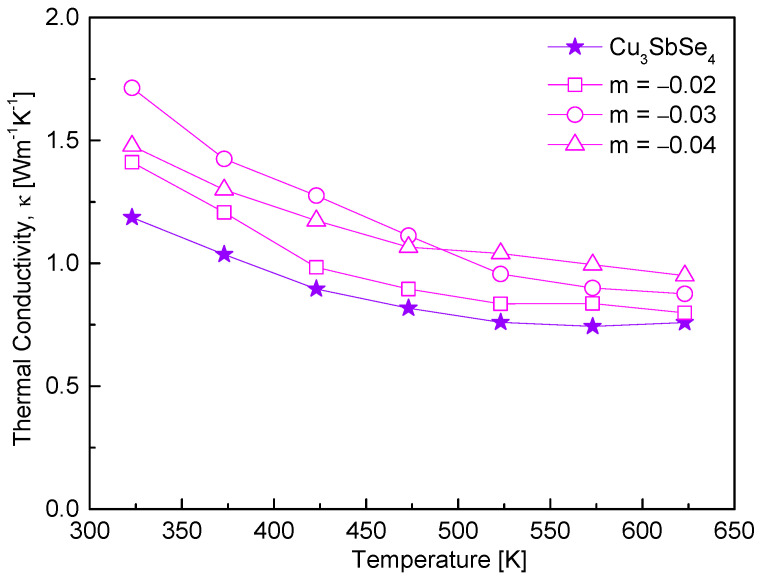
Thermal conductivity of non-stoichiometric permingeatite Cu_3+m_SbSe_4_.

Figure 5 shows the thermal conductivity of Cu_3+m_SbSe_4_. The thermal conductivity values of the non-stoichiometric samples were higher than those of the stoichiometric sample, likely due to the increased carrier concentrations. This leads to enhanced carrier scattering, which results in shorter mean free paths. As the temperature increased in all specimens, the thermal conductivity decreased; no bipolar effect was observed up to 623 K, and phonon−phonon scattering (Umklapp scattering; κ ~ T^−1^) predominated. Non-stoichiometric specimens exhibited thermal conductivities of 1.41–1.71 Wm^−1^K^−1^ at 323 K and 0.95–0.79 Wm^−1^K^−1^ at 623 K, which are higher than those of stoichiometric Cu_3-x_SbSe_4_ (1.19–0.75 Wm^−1^K^−1^ at 323–623 K) [16]. According to Wei et al. [20], Cu_3-x_SbSe_4_ (x = 0–0.075) exhibited a decrease in thermal conductivity with increasing temperature, while as Cu deficiency increased, the thermal conductivity increased from 2.60 Wm^−1^K^−1^ at 323 K for Cu_3_SbSe_4_ to 2.77 Wm^−1^K^−1^ at 323 K for Cu_2.975_SbSe_4_. In contrast, Kumar et al. [22] found a decrease in thermal conductivity with increasing Cu deficiency in Cu_3−δ_SbSe_4_ (δ = 0–0.04) due to more generated defects, ranging from 2.2–1.4 Wm^−1^K^−1^ at 300–675 K for Cu_2.99_SbSe_4_ to 1.9–1.0 Wm^−1^K^−1^ for Cu_2.96_SbSe_4_. According to Kwak and Kim [19], as the Cu content decreased in non-stoichiometric tetrahedrites Cu_12+m_Sb_4_S_13_ (−0.3 ≤ m ≤ 0.3), the thermal conductivity increased from 0.54–0.65 at 323–723 K to 0.97–0.98 Wm^−1^K^−1^. In this study, the increase in thermal conductivity in the Cu-deficient permingeatite is interpreted to be dominantly attributed to the increase in carrier concentration rather than the increase in defect concentration.

**Figure 6:**
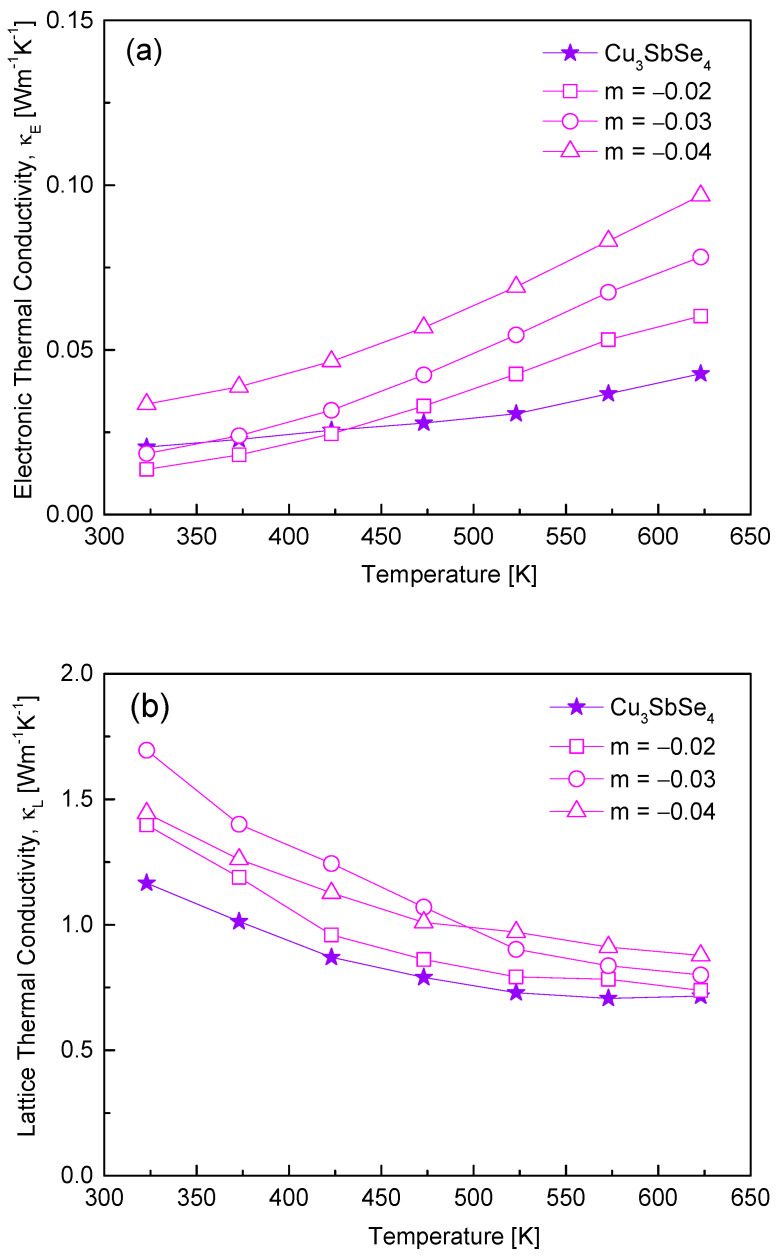
Separation of (**a**) electronic thermal conductivity and (**b**) lattice thermal conductivity of Cu_3+m_SbSe_4_.

In Figure 6, the electronic and lattice thermal conductivities are shown. The thermal conductivity in Figure 5 is determined by heat transfer due to carriers (electronic thermal conductivity, κ_E_) and phonons (lattice thermal conductivity, κ_L_) [23]. In this study, the electronic thermal conductivity was derived using the Wiedemann−Franz law (κ_E_ = LσT), where L is the Lorenz number, and the lattice thermal conductivity was calculated using the equation κ_L_ = κ − κ_E_ [23]. As the temperature and Cu deficiency increased, the electronic thermal conductivity increased, as shown in Figure 6a. This was because the temperature and Cu deficiency increased the carrier concentration. From 323 to 623 K, Cu_3_SbSe_4_ exhibited κ_E_ = 0.02–0.04 Wm^−1^K^−1^, while Cu_2.96_SbSe_4_ showed an increased electronic thermal conductivity, κ_E_ = 0.03–0.10 Wm^−1^K^−1^. In Figure 6b, Cu_3_SbSe_4_ had κ_L_ = 1.17–0.71 Wm^−1^K^−1^ at 323–623 K, while the lattice thermal conductivity of non-stoichiometric permingeatite increased to κ_L_ = 1.38–1.69 Wm^−1^K^−1^ at 323 K and κ_L_ = 0.73–0.95 Wm^−1^K^−1^ at 623 K. In this study, the deficiency of Cu in permingeatite was found to be ineffective in phonon scattering.

**Figure 7:**
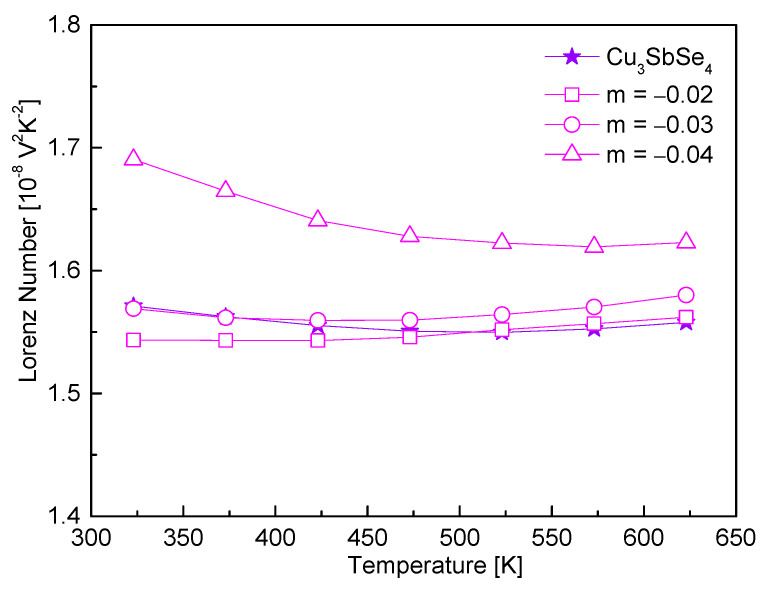
Lorenz number of non-stoichiometric permingeatite Cu_3+m_SbSe_4_.

Figure 7 represents the Lorenz numbers determined using a simple relationship [16], L = 1.5 + exp(−|α|/116). Non-stoichiometric samples with m ≥ −0.03 exhibited Lorenz numbers that were similar to or slightly increased compared to the stoichiometric permingeatite, (1.54–1.60) × 10^−8^ V^2^K^−2^ at 323 K and (1.56–1.58) × 10^−8^ V^2^K^−2^ at 623 K, while the sample with m = –0.04 showed increased Lorenz numbers at 323–623 K, ranging from 1.69 × 10^−8^ to 1.62 × 10^−8^ V^2^K^−2^. The Lorenz number typically ranges from (1.44–2.45) × 10^−8^ V^2^K^−2^, and higher L values are indicative of degenerate semiconducting or a metallic state. Thus, the Cu vacancies in permingeatite served as more evidence of increased carrier concentration.

**Figure 8:**
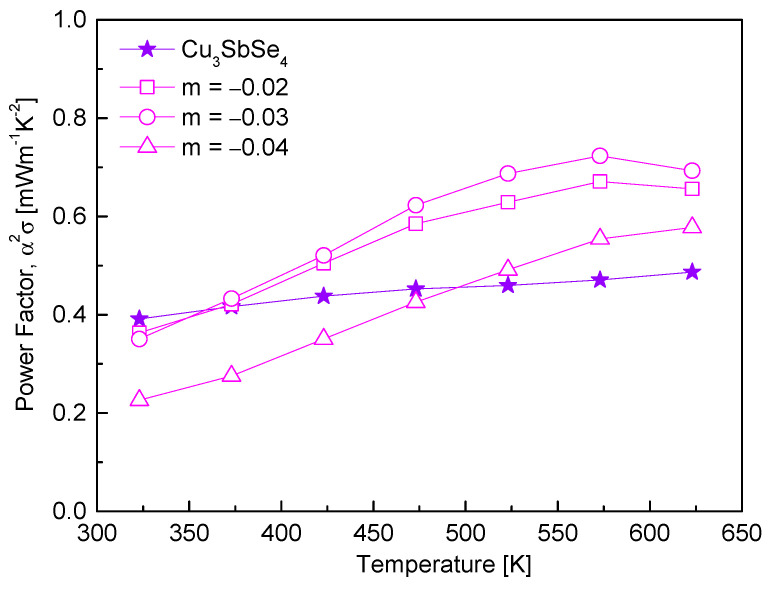
Power factor of non-stoichiometric permingeatite Cu_3+m_SbSe_4_.

The changes in the power factor of Cu_3+m_SbSe_4_ are shown in Figure 8. As the temperature increased, the power factor increased for all specimens, resulting from the temperature dependence of electrical conductivity and the Seebeck coefficient. Although the Seebeck coefficient decreased due to Cu deficiency at a certain temperature (Figure 4), the electrical conductivity increased (Figure 3), resulting in an increase in power factor. For the specimen Cu_2.97_SbSe_4_, a maximum power factor of 0.72 mWm^−1^K^−2^ was recorded at 573 K. This value represents an improvement of approximately 53% compared to the power factor of stoichiometric Cu_3_SbSe_4_ (0.47 mWm^−1^K^−2^ at 573 K). Wei et al. [20] reported that Cu_2.950_SbSe_4_ and Cu_2.925_SbSe_4_ achieved power factor values exceeding 60% higher than that of stoichiometric permingeatite, reaching 0.90 mWm^−1^K^−2^ at 523 K. According to Kwak and Kim [19], Cu-poor tetrahedrites in the off-stoichiometric Cu_12+m_Sb_4_S_13_ (−0.3 ≤ m ≤ 0.3) exhibited higher power factor values compared to Cu-rich specimens, with a maximum value of 1.08 mWm^−1^K^−2^ at 723 K.

**Figure 9:**
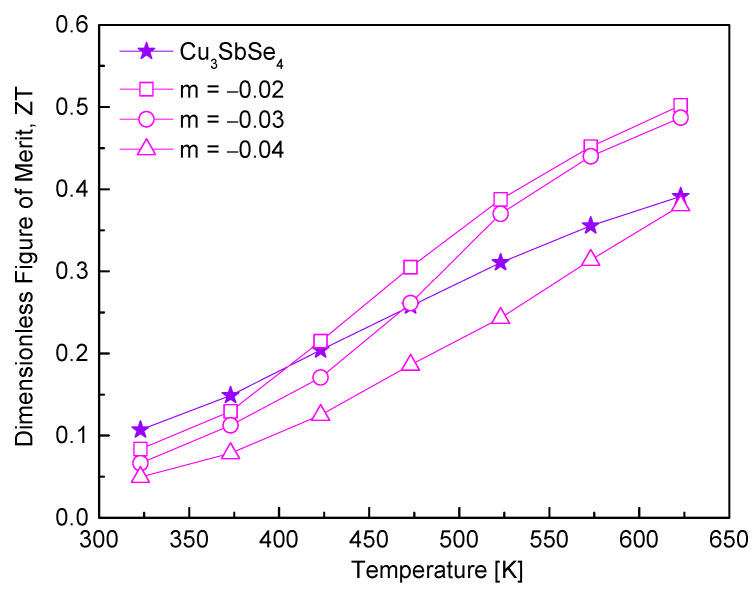
Dimensionless figure-of-merit for non-stoichiometric permingeatite Cu_3+m_SbSe_4_.

The ZT values of Cu_3+m_SbSe_4_ are presented in Figure 9. The ZT value is proportional to the operating temperature of a material; however, it is often constrained by the maximum temperature achievable during fabrication, which is deemed to have the optimal nominal composition for high performance and stability. The incorporation of Cu deficiency could enhance the thermoelectric performance of permingeatite due to increased carrier concentration. Despite increased thermal conductivity, the rise in power factor was significant; thus, for the samples in the range of −0.03 ≤ m ≤ −0.02, the ZT was improved at high temperatures. The highest ZT of 0.50 was recorded at 523 K for Cu_2.98_SbSe_4_. Wei et al. [20] reported a ZT of 0.20 at 673 K for stoichiometric permingeatite Cu_3_SbSe_4_, whereas non-stoichiometric Cu_2.925_SbSe_4_ exhibited a higher ZT of 0.50 at 673 K, suggesting that Cu deficiency helped improve the thermoelectric properties of permingeatite. While their specimens were synthesized using a wet MA process with an alcohol solution as the processing agent in atmosphere gas (95% Ar and 5% H_2_), our specimens were synthesized using a dry MA process without any processing agent in atmosphere gas (100% Ar). Additionally, the synthesized Cu-deficient powders were sintered using the SPS (spark plasma sintering) process at high temperatures (673 K and 703 K), whereas in our study, they were sintered using the HP process at a lower temperature (573 K). Although the influence of Cu deficiency on the thermoelectric properties of permingeatite may be similar, the differences in the synthesis and sintering processes resulted in variations in the magnitude of this influence (i.e., the values of the thermoelectric properties); the highest thermoelectric performance was reported at 673 K in one study, while in our study, the same performance was recorded at a significantly lower temperature of 523 K, which is 150 K lower. Kwak and Kim [19] obtained a ZT of 0.86 at 723 K for stoichiometric tetrahedrite Cu_12_Sb_4_S_13_; however, they recorded the highest ZT value of 0.91 at 723 K for non-stoichiometric Cu_11.9_Sb_4_S_13_. Therefore, it has been confirmed that the deficiencies (vacancies) of Cu in p-type Cu-based chalcogenide compounds increase the charge carrier concentration, thereby helping to improve the thermoelectric performance.

## 4. Conclusions

Cu-deficient permingeatites were prepared by mechanical alloying and hot pressing. Phases, lattice parameters, microstructures, charge transport characteristics, and thermoelectric properties were evaluated depending on the extent of Cu deficiency. No secondary phases were observed, and the relative densities of all samples were above 96.5%. Due to Cu deficiency, the lattice constants significantly decreased, and the tetragonality of permingeatite also decreased. Increased carrier (hole) concentration due to Cu deficiency led to a decrease in the Seebeck coefficient. However, the electrical conductivity increased, resulting in a higher power factor, with Cu_2.97_SbSe_4_ recording a maximum power factor of 0.72 mWm^−1^K^−2^ at 573 K, which is 53% higher than that of stoichiometric permingeatite at the same temperature. While Cu deficiency increased the thermal conductivity, the increase in power factor was more significant, leading to Cu_2.98_SbSe_4_ exhibiting a maximum ZT of 0.50 at 523 K, which is 28% higher than that of stoichiometric permingeatite at the same temperature. Therefore, Cu deficiency in permingeatite enables the improvement of thermoelectric performance through carrier concentration control while maintaining a tetragonal crystal structure and single phase of permingeatite.

## Figures and Tables

**Table 1:** Relative densities, lattice parameters, and charge transport characteristics of stoichiometric and non-stoichiometric permingeatites.

Specimen		Relative Density [%]	Lattice Parameter			Carrier Concentration [10^18^ cm^−3^]	Mobility [cm^2^V^−1^s^−1^]
Nominal	Actual		a [nm]	c [nm]	c/a		
Cu_3_SbSe_4_	Cu_3.14_Sb_0.97_Se_3.89_	98.1	0.5661	1.1280	1.9926	5.2	505
Cu_2.98_SbSe_4_	Cu_2.99_Sb_0.88_Se_4.13_	96.7	0.5655	1.1254	1.9901	7.9	286
Cu_2.97_SbSe_4_	Cu_2.93_Sb_0.83_Se_4.24_	96.5	0.5654	1.1253	1.9903	8.2	210
Cu_2.96_SbSe_4_	Cu_2.91_Sb_0.89_Se_4.20_	98.1	0.5654	1.1253	1.9903	9.6	410

## Data Availability

The original contributions presented in the study are included in the article. Further inquiries can be directed to the corresponding author.
